# THE EQUITABLE AGING IN HEALTH CONCEPTUAL FRAMEWORK: CASE STUDIES OF SOCIAL ISOLATION AND LONELINESS

**DOI:** 10.1093/geroni/igae098.3235

**Published:** 2024-12-31

**Authors:** Angela Perone, Leyi Zhou, Mo’e Yaisikana, Leixuri Urrutia-Pujana

**Affiliations:** University of California Berkeley, Berkeley, California, United States; University of California Berkeley, Berkeley, California, United States; University of California Berkeley, Berkeley, California, United States; University of the Basque Country, Bilbao, California, Spain

## Abstract

Research has well-documented health disparities associated with social isolation and loneliness among older adults. However, much of this research focuses on the United States, and conceptual and theoretical frameworks incorporating power are underdeveloped. This poster presents a new conceptual framework that bridges theories of power and social and political determinants of health to provide a new vision for developing interventions that incorporate power and justice from a global perspective. The Equitable Aging in Health Conceptual Framework foregrounds power as a core driver of social and political determinants of health and the interventions addressing health disparities that flow from power inequities. We argue that interventions that infuse justice must inherently address power, and this conceptual framework aims to help researchers, policymakers, and practitioners accomplish that objective. To illustrate the utility of this conceptual framework, this poster presents three case studies. The first case study applies this conceptual framework to loneliness among rural indigenous older adults in Taiwan. The second case study applies this framework to social isolation among urban community-dwelling older adults in mainland China. The final case study applies this framework to social isolation and loneliness among nursing home residents in Spain. These case studies illustrate how this framework can serve as a tool to visually identify and map how five domains of power can shape social and political determinants of health and a blueprint for conceptualizing interventions that infuse justice.

